# The 5-lipoxygenase pathway: oxidative and inflammatory contributions to the Alzheimer’s disease phenotype

**DOI:** 10.3389/fncel.2014.00436

**Published:** 2015-01-14

**Authors:** Yash B. Joshi, Domenico Praticò

**Affiliations:** Department of Pharmacology and Center for Translational Medicine, Temple University School of MedicinePhiladelphia, PA, USA

**Keywords:** Alzheimer’s disease, amyloid beta, tau, synapse, memory, 5-lipoxygenase, oxidative stress, neuroinflammation

## Abstract

Alzheimer’s disease (AD) is the most common, and, arguably, one of the most-well studied, neurodegenerative conditions. Several decades of investigation have revealed that amyloid-β and tau proteins are critical pathological players in this condition. Genetic analyses have revealed specific mutations in the cellular machinery that produces amyloid-β, but these mutations are found in only a small fraction of patients with the early-onset variant of AD. In addition to development of amyloid-β and tau pathology, oxidative damage and inflammation are consistently found in the brains of these patients. The 5-lipoxygenase protein enzyme (5LO) and its downstream leukotriene metabolites have long been known to be important modulators of oxidation and inflammation in other disease states. Recent *in vivo* evidence using murine knock-out models has implicated the 5LO pathway, which also requires the 5LO activating protein (FLAP), in the molecular pathology of AD, including the metabolism of amyloid-β and tau. In this manuscript, we will provide an overview of 5LO and FLAP, discussing their involvement in biochemical pathways relevant to AD pathogenesis. We will also discuss how the 5LO pathway contributes to the molecular and behavioral insults seen in AD and provide an assessment of how targeting these proteins could lead to therapeutics relevant not only for AD, but also other related neurodegenerative conditions.

## Alzheimer’s disease: background

Alzheimer’s disease (AD) is the most common aging-associated neurodegenerative condition with dementia, marked by profound and irreversible memory impairment and cognitive deficits. The total number of individuals with AD worldwide is estimated to be over 35 million with a predicted estimated annual economic burden of $600 billion USD (Alzheimer’s Association, 2014). The vast majority of AD cases are sporadic, without a clear genetic component, and symptoms of AD typically declare themselves after the age of 65, with 11% of those 65 and older and 32% of 85 and older showing signs of AD (source: Alzheimer’s Association). Current population demographics suggest that those aged 65 and older will increase from 13% now to 20% of total population in 2030, making the future burden of AD a tremendous public health challenge. In stark contrast to the looming public health challenge of AD, current therapeutic options for AD are limited. Although a plethora of agents are currently being investigated in phase II and phase III trials, currently approved medications include several acetylcholinesterase inhibitors and N-methyl D-aspartate (NMDA) antagonists, which do little to modify disease course (Caraci et al., 2013; Tan et al., 2014). Given the confluence of an increased burden of AD in the near-future to health systems globally and a lack of approved therapeutic targets, investigation of targets that address multiple different facets of AD pathophysiology must be actively sought to help address this problem. Below we will give an overview of some of the molecular insults associated with AD, and discuss how the 5-lipoxygenase (5LO) enzyme presents a novel molecular pathway that is an attractive target for AD therapy.

## Aβ and tau in Alzheimer’s disease

Through extensive clinical and molecular work over the past several decades, the biochemical pathways that lead to AD pathology have been well characterized. The cardinal pathologies observed in AD are the extracellular deposits of amyloid-β protein (Aβ) known as Aβ plaques, and intracellular accumulations of the hyperphosphorylated microtubule-associated tau protein known as neurofibrillary tangles (Iqbal et al., 2010; Holtzman et al., 2011). Current dogma presumes Aβ as the upstream molecular initiator in AD based on evidence that mutations in the Aβ precursor protein (APP) and presenilins, main components of the pathways that cleave it to produce Aβ peptides, are found in early-onset, familial variants of AD. Additionally, patients with Down’s syndrome, in which there is an additional chromosome 21, the locus of the APP gene, have significantly increased rates of AD when compared with the general population (Wilcock and Griffin, 2013). However, more recent clinical data have also found mutations in the APP gene that are protective and reduce AD risk (Jonsson et al., 2012, 2013).

**Figure 1:**
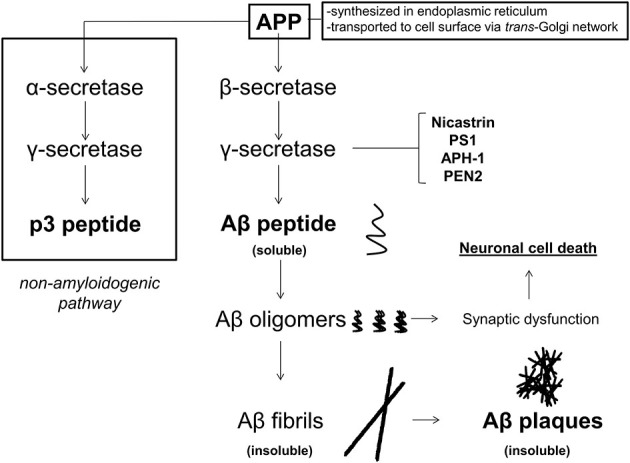
**APP metabolism in Alzheimer’s disease (AD)**. Amyloid β precursor protein (APP) is synthesized in the endoplasmic reticulum and transported to the cell surface through endosomes via the trans-Golgi network. At the cell membrane, APP may undergo either non-amyloidogenic processing or pro-amyloidogenic processing. If APP undergoes non-amyloidogenic processing, it is first cleaved by the α-secretase, and then the γ-secretase to produce p3 peptide, which does not form amyloid deposits. If APP is cleaved by β-, and then γ-secretase (composed of nicastrin, presenilin 1 [PS1], anterior pharynx defective-1 protein [APH-1], and presenilin enhancer 2 [PEN2]), then Aβ peptides are produced. Amyloid-β peptides form oligomers, and then fibrils, which become insoluble, and eventually deposit into Aβ plaques. While initially it was thought that Aβ plaques were the causal pathology in AD, soluble low-n oligomers are currently thought to play the initiating role in synaptic dysfunction and neuronal cell death.

Amyloid-β peptide is formed by the sequential cleavage of APP by the β-secretase (β APP cleavage enzyme, BACE 1) and the γ-secretase complex (composed of the nicastrin, presenillin, PS1), anterior-pharynx defective-1 protein [APH-1], and presenillin enhancer protein [Pen-2], as shown in Figure 1. While APP may be cleaved by α-secretase and then γ-secretase to produce non-amyloidogenic products, the Aβ producing pathway is thought to be priviledged in AD. Generation of higher amounts and subsequent aggregation of Aβ peptide through the sequential β- and γ-secretase cleavages is thought to lead to soluble oligomers, followed by longer fibrils, and finally insoluble plaques, which are found abundantly in the vast majority of AD patients on autopsy. Although insoluble plaques have been found in the brains of patients without AD, current thinking is that low-*n* Aβ oligomers perpetuate the brunt of molecular insults in AD rather than insoluble plaques *per se* (Ono and Yamada, 2011).

**Figure 2:**
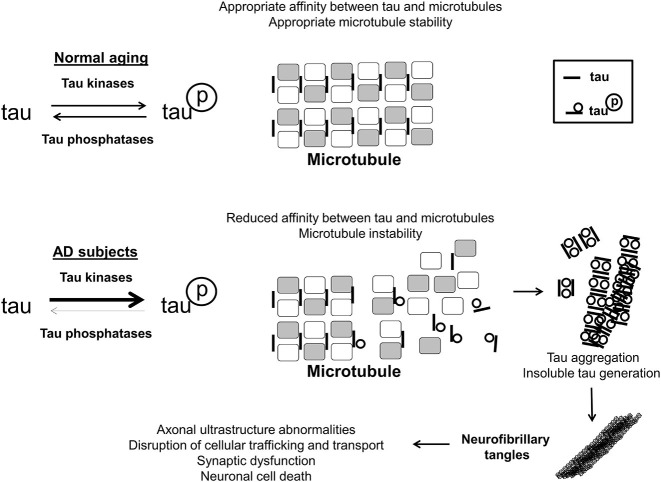
**Tau phosphorylation in AD**. In the brains of aged, disease-free control subjects, tau is associated with, and has affinity for microtubules, stabilizing them, and promoting normal axonal functioning. Tau may be phosphorylated and de-phosphorylated, with phosphorylation altering its microtubule affinity and stabilizing function, and this is maintained through an interplay of tau-associated kinases and phosphatases. In the brains of AD subjects, tau becomes hyperphosphorylated by means of relatively greater net tau kinase activity/reduced phosphatase activity. Hyperphosphorylated tau has a much lower affinity for microtubules, resulting in disruption of appropriate microtubule structure. Additionally hyperphosphorylated tau aggregates together, eventually generating insoluble tau species which eventually form neurofibrillary tangles intracellularly. As a result of neurofibrillary tangle formation, cellular trafficking and transport is perturbed, leading to cell death and synaptic dysfunction.

The hyperphosphorylation of the microtubule-associated tau protein also contributes to the molecular damage in AD. Tau is thought to be important in neuronal ultrastructure and axonal transport, both critical to overall neuron function and signaling (Iqbal et al., 2010). Upon hyperphosphorylation, tau loses affinity for microtubules, dissociating from them, and begins to aggregate, eventually precipitating inside neuronal cells, as shown in Figure 2. While Aβ is hypothesized to be the initiating event, cortical burden of neurofibrillary tau tangles correlates with dementia severity much more robustly (Oddo et al., 2006; Nelson et al., 2007). Normal tau protein phosphorylation status is generally thought to be maintained by the relative balance of tau-specific kinases(s), which would add phosphate, and phosphatase(s), which would remove phosphate. At present, cyclin-dependent kinase 5 and glycogen synthase kinase 3 beta represent two such tau kinases that have been found to be abnormally functional in the brains of AD patients, and therefore of functional importance (Hanger et al., 1992; Baumann et al., 1993; Pei et al., 1999).

Although the specific mechanisms from disruption of normal functioning of both Aβ and tau to AD symptomatology remains unclear, both have been associated with oxidative stress and inflammation found in the brains of AD patients.

## Oxidative stress and inflammation in Alzheimer’s disease

Balance of oxidation and reduction is critical to appropriate cellar function and results from the interplay of mechanisms that produce pro-oxidant molecules and those processes that detoxify them. The brain receives an overwhelming proportion of total body blood flow (i.e., oxygen) and glucose when adjusted for its weight, and in neurons, this relatively high oxygen and glucose requirement is directed towards energy generation (i.e., ATP production) and oxidative phosphorylation in mitochondria (Scheinberg and Stead, 1949; Smith et al., 2007). The vast majority of oxidative species (predominantly reactive oxygen species such as superoxide anions, hydroxyl radicals and hydrogen peroxide) produced by this metabolism is detoxified by antioxidant vitamins (e.g., vitamin C, and E) as well as superoxide dismutase, catalase and glutathione peroxidase (Adibhatla and Hatcher, 2010). A basal level of oxidants contributes to appropriate cellular signaling and modulates cell function but elevated levels of reactive oxygen species leads to oxidation and irreversible modification of proteins, lipids and nucleic acids which ultimately result in the disruption of their regular function (Ray et al., 2012). Interestingly, consistent evidence supports the hypothesis that a progressive accumulation of oxidative stress damage to important cellular molecules is a fundamental mechanism involved in the process of aging, which is the strongest risk factor for developing sporadic AD (Jacob et al., 2013).

In AD, oxidative modifications of proteins, lipids, DNA in the brain have been repeatedly found. Thus several studies have shown the presence of DNA and RNA oxidation products such as 8-oxo-2′-deoxyguanosine (8-oxo-dG) and 8-dihydro-2′-guanosine (8OH) in AD brains, and lipid peroxidation products such as F2-isoprostanes and various reactive aldehydes have been reported in not only in AD brains but also in patient cerebrospinal fluid of patients with a clinical diagnosis of AD (de Leon et al., 2007; Sutherland et al., 2013). Although the precise mechanism of oxidation stress in AD remains elusive, the sulfur atom of methionine 35 in Aβ peptide has been shown to produce free radicals (Butterfield et al., 2013). Replacement of methionine to cysteine reduced oxidative damage in model organisms such as* C. elegans*, and substitution of sulfur with a methylene moiety reduces oxidation *in vitro* of the Aβ peptide (Yatin et al., 1999; Dai et al., 2007). Although clinical trials have not yet found a meaningful disease-modifying effect of antioxidant agents in the treatment of AD, many studies have founds significant association between diets rich in antioxidants and lower risk of AD-risk (Pocernich et al., 2011).

Besides oxidative stress damage, altered inflammatory reactions are strongly associated with AD pathology and cognitive dysfunction. The dysregulation of inflammatory cytokines as well as immune cells (i.e., microglia and astrocytes) activation in AD brains has been well-documented. Microglia have some ability to clear Aβ, but are unable to effectively phagocytate high concentrations (or insoluble conformations) of it resulting in aberrantly activated microglia that associate with both Aβ plaques and neurofibrillary tangles (Hickman et al., 2008; Johnston et al., 2011; D’Andrea et al., 2004; Krabbe et al., 2013; Morales et al., 2013). Astrocytes can be directly stimulated by Aβ to secrete pro-inflammatory molecules, and evidence is growing that they have the ability to produce Aβ peptides themselves (Blasko et al., 2000; Wang et al., 2011a; Jo et al., 2014). Microgliosis and astrocytosis follow deposition of Aβ plaques and neurofibrillary tangles as shown by immunohistochemistry, and have been shown to precede neuronal loss (Sheng et al., 1997a,b; Sheffield et al., 2000; Wright et al., 2013). Resulting directly from immune cell activation, AD brains also have higher tissue levels of cytokines, including various interleukins (IL) such as IL-1β, tumor necrosis factor α (TNFα), and interferon γ (IFNγ), all independently-linked to increased production of Aβ and tau phosphorylation (Zilka et al., 2012).

Despite this compelling evidence, prospective clinical trials targeting oxidative stress and inflammation have previously not found clear and incontrovertible disease-modifying effects on the progression of AD. With regard to oxidation, the largest prospective clinical trials have tested combinations of the monoamine oxidase inhibitor, selegiline and alpha-tocopherol (i.e., vitamin E) or the cholinesterase inhibitor, donepezil and alpha-tocopherol, either in patients with severe disease or in the prodromal stage, in those with mild cognitive impairment (Sano et al., 1997; Petersen et al., 2005). In these studies, no difference was found between antioxidant-treated groups and controls in terms of disease progression or on cognitive assessment at the end of intervention. Other studies using a variety of antioxidants such as resveratrol and curcumin, among others, have noted either no benefit, or benefit with limited effect sizes in small cohorts (for an excellent review see Mecocci and Polidori, 2012). Recently, the Trial of Vitamin E and Memantine in Alzheimer’s Disease (TEAM-AD) has reported reduction in functional cognitive decline in those receiving alpha-tocopherol compared to placebo (Dysken et al., 2014). However, these results have been criticized due to the relatively high dose of vitamin E used, and the fact memantine alone or in combination with vitamin E did not produce similar protective effects (Corbett and Ballard, 2014).

The earliest anti-inflammatory strategies in AD resulted from data showing reduced AD incidence in patients with rheumatoid arthritis, who have a high exposure to non-steroidal anti-inflammatory agents (McGeer and Rogers, 1992; Stewart et al., 1997). This finding was reproduced in many population studies (for a review, see McGeer and McGeer, 2013). However, placebo-controlled trials for AD using anti-inflammatory agents showed little benefit and significant adverse effects leading to subject dropout, although it is to be noted that the majority of these trials used a relatively short treatment window before trial termination or cessation (McGeer et al., 1996). Despite these trial failures, new data indicating mutations in Triggering receptor expressed on myeloid cells 2 gene (*TREM2*), which encodes a membrane protein found on immune cells, confers significant risk in the development of AD, has reinvigorated interest in an anti-inflammatory strategy in AD (Jonsson et al., 2013).

In recent years, mounting evidence has indicated that Aβ and tau pathologies begin depositing and impair neuronal function long before symptoms are manifest. Give this insight it is unlikely that recent and previously conducted clinical trials can adequately address the true effectiveness of anti-oxidant and anti-inflammatory agents. Additionally, the multifactorial nature of the molecular insults in AD make it likely that a strategy that addresses not only pathological Aβ and tau accumulation, but also oxidation and inflammation would have the best chance of success.

## The 5-lipoxygenase pathway

**Figure 3:**
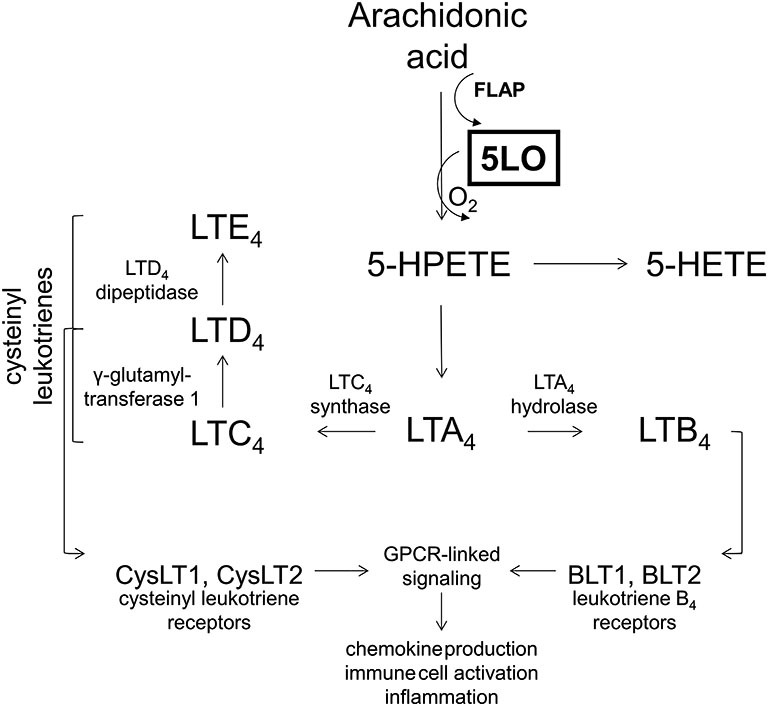
**The 5-lipoxygenase pathway**. The 5-lipoxygenase protein (5LO) inserts molecular oxygen into free and esterified fatty acids with the aid of 5-lipoxygenase activating protein (FLAP). 5-lipoxygenase acts on arachidonic acid to produce 5-HPETE, which is metabolized into 5-HETE or leukotriene A4, depending on the cellular milieu. Leukotriene A4 is then either acted on by LTA4 hydrolase to produce LTB4 or by LTC4 synthase to produce LTC4. Leukotriene B4 may then act on leukotriene B4 receptors (BLT1, BLT2) which are G-protein coupled receptors (GPCRs) to initiate second messenger systems and produce cellular effects. Leukotriene C4 may be acted on by γ-glutamyltransferase 1 to produce LTD4, which may be acted on by LTD4 dipeptidase to yield LTE4. Collectively LTC4, LTD4, and LTE4 are known as the cysteinyl leukotrienes. These cysteinyl leukotrienes all may act on cysteinyl leukotriene receptors (CysLT1, CysLT2) which are also GPCRs to exert downstream effects. Collectively, leukotriene receptor activation is known to modulate chemokine production, immune cell activation and inflammation.

The 5LO inserts molecular oxygen into the 5th carbon of free or esterified fatty acids, most notably arachidonic acid. In order to carry out the reaction, 5LO also requires 5LO activating protein, FLAP, which presents the substrate for enzymatic action (for a complete review on 5LO biology, see Rådmark and Samuelsson, 2010). Immediate products of 5LO include unstable 5-hydroperoxyeicosatetraenoic acid which is either reduced to 5-hydroxyeicosatetraenoic acid or leukotriene A_4_ (LTA_4_). Depending on the cellular milieu, LTA4 can be metabolized either to leukotriene B_4_ or C_4_ (LTB_4_ or LTC_4_), with LTC_4_ further being metabolized to LTD_4_ and LTE_4_ (Bishayee and Khuda-Bukhsh, 2013), as shown in Figure 3.

5-lipoxygenase is found in vasculature, in endothelial cells, as well as throughout the central nervous system, in both neuron and glia (Chu and Praticò, 2009). Interestingly, 5LO expression and its metabolites increase with age in both animal models as well as human subjects. 5-lipoxygenase protein levels are particularly enriched in both cortex as well as hippocampus, areas known to be particularly vulnerable in neurodegeneration, and in AD in particular (Lammers et al., 1996; Chinnici et al., 2007). 5-lipoxygenase promotes lipid peroxidation *in vitro* as well as in brain tissue (Czubowicz et al., 2010; Czapski et al., 2012). Leukotrienes, metabolic products of 5LO activation, initiate immune cell chemotaxis and are critical molecular players in the inflammatory pathophysiology of asthma and allergy (Kanaoka and Boyce, 2014).

## 5LO, FLAP and the Alzheimer’s disease phenotype

Post-mortem studies have initially shown that 5LO is increased in AD (Firuzi et al., 2008; Ikonomovic et al., 2008). A small pilot study in humans has linked 5LO gene polymorphisms to early- and late-onset AD, although large-scale population studies are yet to confirm these findings (Qu et al., 2001). In neuro2A cells harboring the Swedish APP mutation, knockout of 5LO reduces reduces their ability to produce and release soluble Aβ levels (Chu and Praticò, 2011a). This effect is due to reduced activity of γ-secretase, with 5LO knockout lowering steady-state expression of nicastrin, PS1, APH-1 and Pen-2 proteins without affecting either APP, the β-secretase or α-secretase pathway. On the other hand, overexpression of 5LO produces the opposite effect *in vitro*: Aβ levels are elevated, and associated with increased protein and mRNA levels of all γ-secretase proteins. While several molecules have been linked to the regulation of γ-secretase mRNA level, 5LO modulation occurs through the phosphorylation of cyclic adenosine response element-binding protein, CREB. Pharmacological inhibition of 5LO also reproduces 5LO knockout effects (Chu and Praticò, 2011b). Although γ-secretase produces Aβ peptides, which are pathologic in AD, it is also involved in the processing of Notch, a protein critical for neuronal functioning and differentiation (Imbimbo and Giardina, 2011). Clinical trials investigating γ-secretase inhibitors for use in AD have to date been largely unsuccessful because they are hypothesized to have altered Notch signaling as well as Aβ production (Doody et al., 2013). Fortunately, with 5LO modulation, γ secretase-dependent Notch production is unperturbed, making any future and potential use of this class of drug a feasible alternative to calssical γ-secretase inhibitors. By directly controlling the substrate availability for 5LO, FLAP modulation similarly affects Aβ production with FLAP knockout and pharmacological inhibition reducing Aβ through a CREB-mediated γ-secretase down-regulation (Chu and Praticò, 2012). *In vitro* data in neuronal cells have been reproduced in mouse models of AD-like amyloidosis with similar effects of 5LO and FLAP on Aβ, which includes immunohistochemical evidence of plaque reduction upon 5LO or FLAP knockout (Giannopoulos et al., 2013, 2014). Recently, these data have also been reproduced in TgCRND8 animals, with reduction found in amyloid-associated angiopathy upon administration of MK886, an inhibitor of FLAP (Hawkes et al., 2014). Intriguingly, leukotriene metabolites of 5LO have also been reported to increase β- and γ-secretase-mediated generation (Wang et al., 2013).

In addition to Aβ, 5LO and FLAP also modulate tau phosphorylation. In cells overexpressing 5LO, tau is hyperphosphorylated, generating both early-stage, as well as advanced-stage tau phosphoepitopes (Chu et al., 2013). Beyond tau phosphorylation, 5LO overexpression results in production of paired helical filaments of tau, precursors to insoluble tau deposition and neurofibrillary tangle formation. Knockout or inhibition of 5LO or FLAP produces results in amelioration of tau hyperphosphorylation (Chu and Praticò, 2013; Chu et al., 2013). Both *in vivo* in murine models and *in vitro*, 5LO pathway changes are mediated by 5LO and FLAP influence on cyclin-dependent kinase 5 activity (Chu and Praticò, 2013). As mentioned previously, abnormalities in Aβ metabolism are presumed to be upstream of tau phosphorylation but 5LO effects on tau phosphorylation seem to be independent of Aβ. In a series of *in vitro* experiments, γ-secretase blockade in the presence of 5LO overexpression did not prevent 5LO-mediated hyperphosphorylation of tau (Chu et al., 2013). These *in vitro* results have also been corroborated in not only a mouse model of amyloidosis such as the Tg2576 mouse, but also a model with both Aβ and tau pathology such as the 3xTg mouse (Chu et al., 2012b, 2013).

Beyond neuropathology, knockout of 5LO or FLAP mitigates age-dependent AD learning and memory insults seen in two models of AD. In both Tg2576 and 3xTg mice, 5LO knockout or inhibition is associated with improvements in learning and memory over baseline in fear conditioning paradigms while overexpression of 5LO is associated with greater cognitive insult (Chu and Praticò, 2011a; Chu et al., 2012a,b; Giannopoulos et al., 2013, 2014). Knockout or inhibition of 5LO and FLAP also ameliorates markers of synaptic protein pathology and restores hippocampal long-term potentiation to wild-type levels (Giannopoulos et al., 2013, 2014). Even in aged transgenic mice, when significant AD pathology has been deposited, targeting the 5LO pathway appears to improve the overall AD-like phenotype (Di Meco et al., 2014).

## 5LO, FLAP and Alzheimer’s disease-associated oxidation and inflammation

While 5LO and FLAP appear to reduce the cardinal pathologies in AD, they also act on AD pathology-induced oxidative and inflammatory insult.

In cultured rat hippocampal neurons, 5LO pathway inhibition results in reduced Aβ-induced reactive oxygen species generation and subsequent calcium dysregulation in a concentration-dependent manner (Goodman et al., 1994). 5-lipoxygenase pathway inhibition also protects against glutamate-induced excitotoxicity *in vivo* in rats, particularly in aged animals (Uz et al., 1998). In other *in vitro* systems, 5LO overexpression does not by itself lead to oxidative damage, but when overexpressed in the presence of Aβ peptides, reduces glutathione peroxidase and catalase levels (Wang et al., 2011b). 5-lipoxygenase pathway inhibition by the pyrazole CNB-001, a 5LO-specific inhibitor, protects against endoplasmic reticulum dysfunction and proteasome toxicity induced by Aβ both in cultured neurons as well as *in vivo* (Valera et al., 2013). Interestingly, inhibition of FLAP is not sufficient to protect against Aβ oxidative toxicity *in vitro*, which suggests that even when 5LO is decoupled from Aβ metabolism, its ability to insert molecular oxygen is preserved, retaining pro-oxidative properties in a leukotriene-independent fashion. However, this phenomenon is otherwise not well described or replicated in neurons and requires further scrutiny and exploration. On the other hand, leukotriene blockade has been linked to reduction in cognitive deficits induced by traumatic brain injury in rats, a source of oxidative stress, and a well-known risk factor for AD (Corser-Jensen et al., 2014).

Besides oxidation, both 5LO and FLAP are active players in the neuroinflammation found in AD. Disruption of the 5LO pathway, either genetically or pharmacologically, reduces not only microglia, but also astrocytosis in the brains of AD animals, seen on immunohistochemical analyses (Chu and Praticò, 2012). Moreover, with 5LO/FLAP disruption there is an associated reduction in pro-inflammatory cytokine levels. An argument can be made that reduction in Aβ and tau pathology caused by 5LO/FLAP inhibition independently predisposes AD transgenic animals to have reduced neuroinflammation at baseline. While this may be true to some extent, chronic lipopolysaccharide administration in AD animals lacking 5LO increases steady-state levels of γ-secretase machinery and tau phosphorylation but does not change baseline microgliosis, astrocytosis, or brain levels of inflammatory cytokines (Joshi et al., 2014). This line of data suggests that the 5LO system’s contribution to the neuroinflammation does not depend exclusively on Aβ or tau.

## Implications for Alzheimer’s disease therapy and beyond

Although several clinical trials have targeted Aβ specifically, increasingly, their failures have lead commentators to reevaluate not only the time course of intervention in AD, but also the selected therapeutic targets. While tau is increasingly being recognized as a viable target, and anti-oxidant and anti-inflammatory strategies are frequently employed in studies to modify disease course, no agent to date has been employed that independently modulates Aβ metabolism, tau phosphorylation, oxidation and inflammation. Although more work must be done to dissect the mechanisms of 5LO action in model systems more advanced than mice before considering it as a viable target, 5LO pathway suppression has the added benefit of being safe in other chronic conditions such as asthma (i.e., Zileuton, Monteleukast). Beyond AD, 5LO pathway targeting may also be useful in other dementias and related conditions including: non-AD amyloidoses (i.e., cerebral amyloid angiopathy), tauopathies (e.g., frontotemporal dementia, chronic traumatic encephalopathy, progressive supranuclear palsy), oxidation-linked nervous system disorders (i.e., amyotrophic lateral sclerosis) and inflammation-centered disease processes (i.e., multiple sclerosis). Further work exploring the 5LO system would undoubtedly shed more light on the molecular pathways common to neurodegeneration and represent a novel and promising platform for future drug development.

## Conflict of interest statement

The authors declare that the research was conducted in the absence of any commercial or financial relationships that could be construed as a potential conflict of interest.
